# The Bioactivity and Phytochemicals of *Muscari comosum* (*Leopoldia comosa*), a Plant of Multiple Pharmacological Activities

**DOI:** 10.3390/ijms25052592

**Published:** 2024-02-23

**Authors:** Varun Jaiswal, Hae-Jeung Lee

**Affiliations:** 1 Department of Food and Nutrition, College of BioNano Technology, Gachon University, Seongnam 13120, Republic of Korea; skysea@gachon.ac.kr; 2 Institute for Aging and Clinical Nutrition Research, Gachon University, Seongnam 13120, Republic of Korea; 3 Department of Health Sciences and Technology, GAIHST, Gachon University, Incheon 21999, Republic of Korea

**Keywords:** pharmacological activities, cancer, phytochemicals, *Muscari comosum*, obesity, diabetes, antioxidants, *Leopoldia comosa*

## Abstract

*Leopoldia comosa* (LC), popularly known as *Muscari comosum*, spontaneously grows in the Mediterranean region and its bulbs are used as a vegetable. Traditionally, they are also used to treat various diseases and conditions, which has inspired the study of the pharmacological activities of different parts of LC. These studies revealed the numerous biological properties of LC including antioxidant, anti-inflammatory, anti-diabetes, anti-obesity, anti-cancer, anti-Alzheimer’s disease, antibacterial, and immune stimulant. High antioxidant activity compared to other non-cultivated plants, and the potential role of antioxidant activity in other reported activities make LC an excellent candidate to be developed as an antioxidant plant against important associated diseases. The presence of a diverse class of phytochemicals (*n* = 85), especially flavonoids and homoisoflavones, in LC, also imparts significance to the nutraceutical candidature of the plant. However, limited animal studies and the lack of a directional approach have limited the further design of effective clinical studies for the development of LC. The current study is the first attempt to comprehensively compile information regarding the phytochemicals and pharmacological activities of LC, emphasize the targets/markers targeted by LC, important in other activities, and also highlight the current gaps and propose possible bridges for the development of LC as a therapeutic and/or supplement against important diseases.

## 1. Introduction

*Leopoldia comosa* (L.) Miller (LC), popularly known as *Muscari comosum*, is a vegetable spontaneously growing in the Mediterranean region, including southern and central Europe, northern Africa, and southern-western Asia [1]. The bulbs of LC are commonly called lampascioni or cipudizze (in Italy) and Bassila (in Morocco) and are known to have been used as food for a long time. People from Egypt, Greece, and the Mediterranean region have been habituated to eating LC bulbs [1,2]. LC also has economic significance, as the trade in the plant bulbs is an important income source for collectors in Morocco through export, especially to Italy [2]. Nowadays, the bulbs are used in a variety of recipes, including boiled bulbs served with sweets; peeled, cut, and fried in olive oil and served with cheese or eggs; boiled after 24 h of soaking in water and dressed with vinegar, oil, salt, and pepper; pickled bulbs in the Benevento region; and in the Murge region, cooked covered in hot ash, and dressed with oil, salt, and pepper after removing the outer layer. Bulbs are also used as an alternative to onions and are sometimes eaten raw but it is not common practice, as cooking is required to neutralize their characteristic bitter taste [1]. Despite various uses, LC is generally not cultivated because it is easy to find it growing in wild conditions.

LC is also a rich source of various phytochemicals, which give its unique taste. Several phytochemicals (*n* = 85) have been reported in LC, mostly identified in bulbs. These diverse phytochemicals belong to different classes, such as phenolic acids, fatty acids, flavonoids, triterpenes, phytosterols, and homoisoflavones. Among the phytochemicals, flavonoids and homoisoflavones are more emphasized for the reported pharmacological activities of the bulbs [3].

Traditionally, LC has been used to treat various diseases and conditions, such as dermatological affections, digestive disorders, toothache, pus discharge from the lungs, and spots; it has also been used as a diuretic and emollient [1,2,4]. Inspired by the traditional uses, the medicinal properties of LC have been investigated in several pharmacological studies, especially in recent years. In these studies, LC was found to have different pharmacological properties including antioxidant, anti-inflammatory, anti-diabetes, anti-obesity, anti-cancer, anti-Alzheimer’s disease (AD), antibacterial, and immune stimulant in various in vitro and in vivo experiments.

The antioxidant activity of LC was investigated in the majority of studies, and it was found to be the highest in screening experiments among other plant extracts [5]. One of the primary reasons for focusing on LC’s antioxidant activity is that its antioxidant properties may also contribute to the majority of other reported LC activities, such as anti-inflammatory, anti-diabetes, anti-obesity, anti-cancer, anti-AD, and immune stimulant [6,7,8,9]. The presence of various phytochemicals, especially polyphenols such as flavonoids and homoisoflavones, in different parts of the plant such as bulbs, leaves, and inflorescence also supports the antioxidant activities of these parts.

Despite its gastronomical importance, high mineral contents, the presence of numerous biologically active phytochemicals, and several potential biological properties, the complete compilation of the phytochemicals and pharmacological activities of LC is still absent in the literature [10]. This may be an important reason for the underutilization of this vegetable. Hence, in the current study, the comprehensive compilation of phytochemicals as well as pharmacological activities of LC has been attempted for the first time, which also highlights the current gaps and proposes possible bridges for the development of LC as a therapeutic and/or supplement against important diseases. Another novel aspect of this study is that it also points out imported drug targets and markers modulated by LC, and emphasizes their importance in other known pharmacological activities to promote them in future research and development studies.

## 2. Literature Search

The systematic literature search was carried out against important scientific literature databases such as Web of Science, Scopus, and PubMed. The keywords utilized to search the databases were *Muscari comosum*, *Leopoldia comosa*, and their combinations with phytochemicals, phytoconstituent, disease, obesity, diabetes, cancer, antioxidant activities, and inflammation and pharmacological activities. The relevant research and review articles, books, book chapters, and other documents from the search results published in English until December 2023 were considered in this study.

## 3. Botanical Description

**Figure 1:**
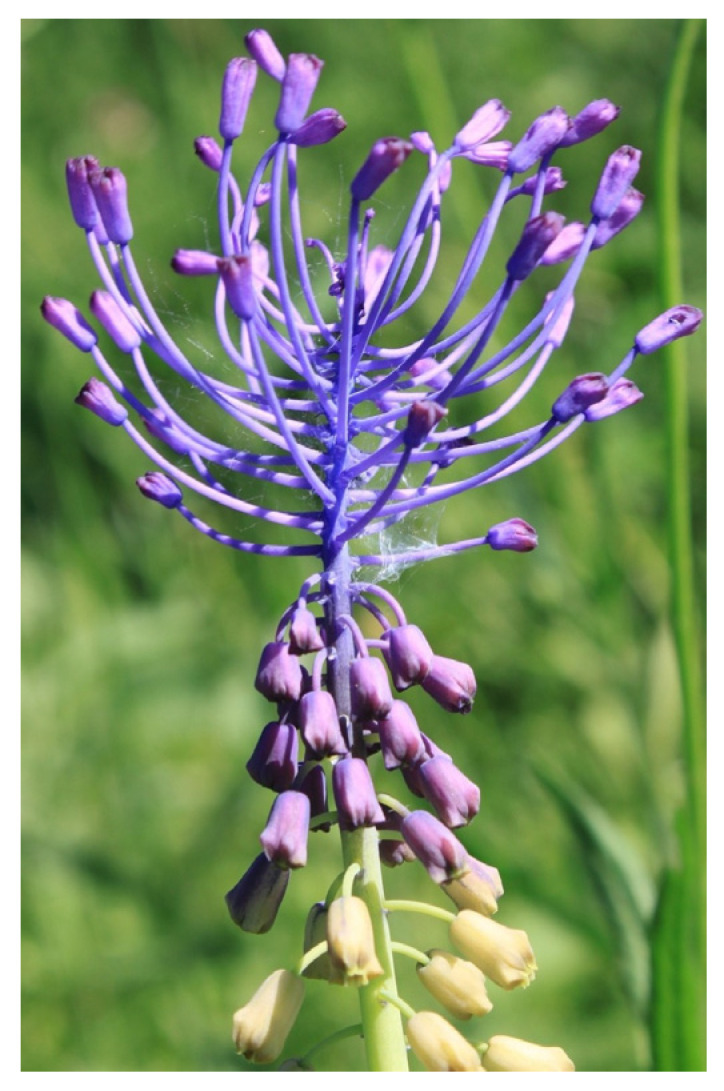
Image of *Leopoldia comosa* (*Muscari comosum*) [image by Emilian Robert Vicol from Pixabay].

LC is a perennial bulbous flowering plant that belongs to the family Asparagaceae (Figure 1). The LC bulbs are pink to reddish, spherical to oval in shape [1]. The leaves of the plant are linear, with a size of around 7–40 × 0.5–1.7 cm. The leaves are mostly shorter than the scape which is 80 cm long. The scapeis erect and has a cylindrical and glabrous shape. The raceme of the plant is also cylindrical in shape; it can be pyramidal and lax. The fertile and sterile flowers are pale brown and bright violet and later become smaller. The fertile flowers are urceolate and patent at the anthesis, with pedicel lengths between 4 and 10 mm. The sterile flowers are from globose to obovoid in shape and they are assembled in a corymbose terminal tuft with pedicels between 2 and 6 mm long, fleshy, ascending, and violet in color. The obovoid-shaped capsule of LC is the size of around 10–15 × 6–8 mm. The pollen grains are monads, monosulcate, ellipsoidal, isopolar, and bilaterally symmetrical. Flowering usually occurs in March and April in the Mediterranean region, and pollination happens through insects [11].

## 4. Toxicity and Adverse Reactions

Limited toxicity studies on LC have been conducted in the literature, as LC bulbs have been used as a vegetable in different populations. The toxicity of the different types of extracts from the bulbs was around 20% in HepG2 cells at up to a 600 μg/mL dose. In the same study, the 70% methanol extract of the bulbs helped in the proliferation of cells in 24-h treatment even at the highest concertation of the extract. However, a reduction in cell viability was observed in 72-h treatment. In a similar study, the extracts of raw and cooked bulbs showed a different inhibition activity against the MCF-7 cell line. The extract from the cooked bulb had an IC_50_ value of 669.3 μg/mL while the extract from the raw bulb had 10.27 μg/mL [12]. In animal experiments, up to 60 mg/kg of the dose has been used comfortably on rats in models as a treatment against obesity [13]. Allergy to LC bulbs is rare: only one case has been reported. In this rare example, IgE-mediated allergies to LC bulbs were observed in a 32-year-old patient after ingestion of a very low quantity of it [14].

## 5. Phytochemicals Reported in LC

Several phytochemicals have been reported in the bulbs and other aerial parts of LC such as leaves and inflorescence (Table 1). Most of these studies were focused on bulbs, as the bulb of the plant is the part that is used as a dietary vegetable. The prime objective behind the phytochemical identification in LC bulbs was the discovery of phytochemicals responsible for the reported biological activities and taste of the plant bulbs. Hence, in several studies, further biological properties were also analyzed after the phytochemical studies. Various important phytochemicals discovered in these studies may explain the reported biological properties of LC.

In the initial study, inspired by the antitumor activity of LC bulbs, the mixture of glycosides from the bulbs was isolated through extraction [15,16]. After the acidic methanolysis, through nuclear magnetic resonance (NMR), one important aglycone moiety was identified as 27-norlanostane triterpene eucosterol [15] (Table 1). The researchers also identified two more compounds (ketotriol and diketotriol), based on the same 27-norlanostane skeleton from the mixture [17] (Table 1). Later, the researchers isolated the nortriterpene fraction from the extract of LC bulbs, and a series of nortriterpenes were identified, including (23*R*)-17,23-epoxy-33,31-dihydroxy-27-nor-5α-lanost-8-ene-15,24-dione and (23*R*)-17,23-epoxy-31-hydroxy-2 7-nor-5α-lanost-8-ene-3,15,24-trione [18,19]. In a series of studies, researchers also isolated and elucidated the structure of various glycosides, i.e., muscarosides A, B, C, D, E, and F [15,20,21,22,23].

Later, the presence of a new class of natural phytochemical homoisoflavonoids (3-benzylidenechroman-4-ones) in LC bulbs prompted research to identify the new compounds of this class. Like glucosides, in a series of studies, the researchers identified compounds of this class including muscomosin, comosin, 8-*O*-demethyl-8-*O*-acetyl-7-*O*-methyl-3,9dihydropunctatin, 3′-hydroxy-3,9dihydroeucomin, 4′demethyl-3,9-dihydroeucomin, 7-*O*-methyl-3,9_dihydropunctatin, 8-*O*-demethyl-7-*O*-methyl-3,9_dihydropunctatin, and 3,9-dihydroeucomnalin in the bulbs [3,24,25]. NMR imaging was used for the spectral characterization of the compounds [24].

The researchers also conducted a study for the identification of compounds that are important for the taste of LC bulbs (taste-active compounds). A series of solvents (hexane, ether, dichloromethane, ethanol, and water) according to polarity was used to extract and identify the compounds present in the bulbs. Among these solvents, ether solvent was used to identify phytochemicals responsible for the taste, as the taste of this fraction was selected by the sensory evaluation team. ^1^H-NMR spectroscopy was used to identify the pure component of the extract separated through column chromatography, followed by preparative thin layer chromatography (TLC). Three identified compounds, muscomin, 8-*O*-demethyl-7-*O*-methyl-3,9-dihydropunctatin, and 3,9-dihydroeucomnalin, were suggested to be the contributors to the taste of LC bulbs in the study. The study also suggested taking these molecules for further experiments for biological activities [26].

In several studies, the initial interest was to analyze the total phenolic and flavonoid content of the extract from LC, as compounds from both chemical classes are considered to be responsible for the biological activities of medicinal plants.

For the first time, the lipophilic profile of the extract from the bulbs of LC was investigated after measuring the total phenolic and flavonoid amounts [27]. To analyze the nonpolar components of the extract, the *n*-hexane extract was utilized for gas chromatography coupled with mass spectrometry analysis (GCMS). The major constituents of the *n-*hexane extract were fatty acids and their ethyl ester, which comprised more than half percent of the total components of the *n-*hexane extract. Among fatty acids, palmitic acid and ethyl palmitate comprised more than 37% of the extract [27].

In the initial studies, the ethanol extract from the bulbs of LC was found to have flavonoid and phenolic contents equivalent to 23.4 mg of quercetin and 56.6 mg of chlorogenic acid per gram of extract [27,28]. Considering the use of bulbs as edible vegetables, the total phenolic and flavonoid content of cooked (traditionally boil-cooked and steam-cooked) was also studied along with raw bulbs [12]. The phenolic and flavonoid contents were found to decrease in both types of cooking. The maximum phenolic content was present in the raw extract (92.47 ± 0.020), and it decreased to 49.80 ± 0.012 and 39.53 ± 0.027 mg chlorogenic acid equivalents (CAE) per g FW in steam-cooked and boiled extract, respectively. Similarly, the maximum flavonoid content was present in the raw extract (4.57 ± 0.003), which decreased to 1.63 ± 0.010 and 0.635 ± 0.026 mg quercetin equivalents (QE) per g in steam-cooked and boiled extract, respectively [12].

Because LC bulbs from both wild and cultivated plants are used as a vegetable, the phenolic and flavonoid contents have been studied in both types. A high difference in phenolic and flavonoid contents was found to be present in the bulbs of cultivated and wild plants. Both phenolic and flavonoid contents were found to be higher in the bulbs of wild plants (264.33 and 10.40 mg/g, respectively), compared with bulbs from the cultivated plants (42 and 5.74 mg/g, respectively) [29]. Afterward, phytochemical profiling to identify the individual phytochemicals present in the extract of the bulbs from both cultivated and wild-grown plants was conducted in the *n-*hexane fraction of the extract through GC-MS analysis. A total of 12 compounds were identified in the bulbs of wild-grown plants and 22 compounds were found to be present in the bulbs of cultivated plants (Table 1). Later, a methanolic (70%) extract of LC bulbs was utilized to study the presence of phytochemicals after total phenolic and flavonoid analysis. The total phenolic and flavonoid contents were found to be 57.67 mg Gallic acid equivalents (GAE)/g and 18.79 mg QE/g in the extract. Further, HPLC analysis compared with external standards revealed the presence of 12 different compounds in the extract. Out of these compounds, seven were phenolic acids and five were flavonoids. The most abundant phenolic acid and flavonoid were p-coumeric acid and catechin, respectively (Table 1) [30].

Researchers have also used the aerial parts of LC such as leaves and inflorescence to analyze phytochemical components. The total phenolic and flavonoid contents of the hydrochloric extract of leaves were found to be 50.50 mg/g and 4.59 mg/g, respectively. Similarly, the total phenolic and flavonoid contents of the hydrochloric extract of inflorescence were found to be 47.67 mg/g and 5.61 mg/g, respectively. The identification of phytochemicals of leaves and inflorescence was carried out on *n-*hexane and dichloromethane fractions of the hydrochloric extract of both leaves and inflorescence, considering the polarity of the phytochemicals present in the extracts. In the *n-*hexane fraction, three unique phytosterols (two in inflorescence and one in leaves) and eighteen unique fatty acids (sixteen in leaves and nine in inflorescence) were identified in GCMS analysis (Table 1). Similarly, in the dichloromethane fraction, nine unique phytocompounds (seven in inflorescence and five in leaves) were identified in the study (Table 1) [31]. Researchers also reported the high content of important minerals in the bulbs of LC. The mineral content of the bulbs was studied through inductively coupled plasma atomic emission spectroscopy, which revealed high contents of important minerals such as iron, potassium, phosphorus, sodium, copper, magnesium, and calcium, i.e., 33,552, 1843.14, 756.36, 439.65, 303.9, 272.37, and 20.55 mg/kg, respectively. Low content (<3 mg) of minerals such as selenium, strontium, and zinc was also found to be present in LC bulbs [32]. Later, researchers prepared different types of aqueous and organic extracts to achieve the optimal concentration of bioactive phytochemical class of compounds such as phenolic, flavonoids, and tannins [32]. Among three aqueous and six organic extracts, the phenolic, flavonoid, and tannin content was found to be maximum in diethyl ether extract, i.e., 129.75 ± 0.29 μg GAE/mg, 988.26 ± 0.18 μg QE/mg, and 30.22 ± 0.15 μg CE/mg, respectively [32]. Recently, aqueous and methanolic (50 and 70%) extracts of LC bulbs were used to study the total phenolic and flavonoid contents before the evaluation of their biological properties. The maximum phenolic and flavonoid contents were found to be present in the 70% methanolic extract, i.e., 58.72 mgGAE/g and 20.37 mg QE/g, respectively [33].

**Table 1:** Different phytochemicals reported in *Leopoldia comosa*.

Sr. No.	Compound Name	Amount (mg/g)	Compound Type	Type of Extract/Fraction	Part of Plant	Ref
1	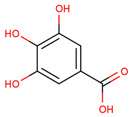 Gallic acid	0.31	Phenolic acid	Methanol extract	Bulbs	[30]
2	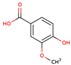 Vanillic acid	0.28	Phenolic acid	Methanol extract	Bulbs	[30]
3	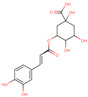 Chlorogenic acid	0.85	Phenolic acid	Methanol extract	Bulbs	[30]
4	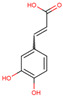 Caffeic acid	0.45	Phenolic acid	Methanol extract	Bulbs	[30]
5	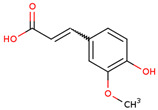 Ferulic acid	1.32	Phenolic acid	Methanol extract	Bulbs	[30]
6	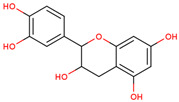 Catechin	1.17	Flavonoids	Methanol extract	Bulbs	[30]
7	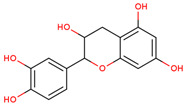 Epicatechin	0.36	Flavonoids	Methanol extract	Bulbs	[30]
8	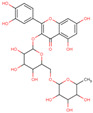 Rutin	0.11	Flavonoid glycoside	Methanol extract	Bulbs	[30]
9	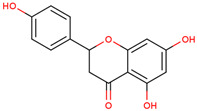 Naringenin	0.17	Flavonoids	Methanol extract	Bulbs	[30]
10	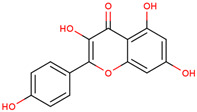 Kaempferol	0.24	Flavonoids	Methanol extract	Bulbs	[30]
11	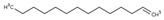 1-Tetradecene	0.04	Hydrocarbon	*n*-hexane fraction of extract from cultivated bulbs	Bulbs	[30]
12	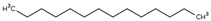 Tetradecane	0.03	Hydrocarbon	*n*-hexane fraction, cultivated bulbs	Bulbs	[29]
13	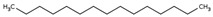 Pentadecane	0.10	Hydrocarbon	*n*-hexane fraction of extract from cultivated bulbs	Bulbs	[29]
14	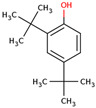 Phenol, 2,4-bis(1,1-dimethylethyl)-	0.13	Other	*n*-hexane fraction of extract from cultivated bulbs	Bulbs	[29]
15	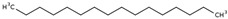 Hexadecane	0.14	Hydrocarbon	*n*-hexane fraction of extract from cultivated bulbs	Bulbs	[29]
16	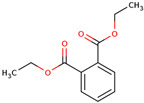 1,2-Benzenedicarboxylic acid, diethyl ester	0.04	Phthalate ester.	*n*-hexane fraction of extract from cultivated bulbs	Bulbs	[29]
17	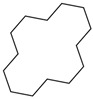 Cyclotetradecane	0.01	Other	*n*-hexane fraction of extract from cultivated bulbs	Bulbs	[29]
18	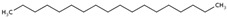 Octadecane	0.13	Hydrocarbon	*n*-hexane fraction of extract from cultivated bulbs	Bulbs	[29]
19	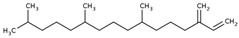 Neophytadiene	0.14	Hydrocarbon	*n*-hexane fraction of extract from cultivated bulbs	Bulbs	[29]
20	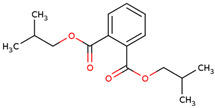 1,2-Benzenedicarboxylic acid, bis(2-methylpropyl) ester	0.32	Other	*n*-hexane fraction of extract from cultivated bulbs	Bulbs	[29]
21	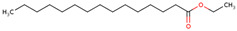 Pentadecanoic acid, ethyl ester	1.27	Fatty acid ester	*n*-hexane fraction of extract from cultivated bulbs	Bulbs	[29]
22	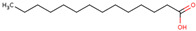 Myristic acid	17.52	Fatty acid	*n*-hexane fraction of extract from wild bulbs	Bulbs	[29]
23	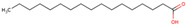 Margaric acid	1.20	Fatty acid	*n*-hexane fraction of extract from cultivated bulbs	Bulbs	[29]
24	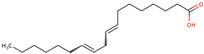 8,11-Octadecadienoic acid	1.34	Fatty acid	*n*-hexane fraction of extract from wild bulbs	Bulbs	[29]
25	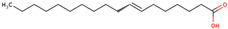 7-Octadecenoic acid	1.30	Fatty acid	*n*-hexane fraction of extract from wild bulbs	Bulbs	[29]
26	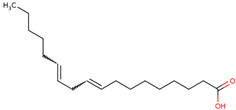 Linoleic acid	12.92	Fatty acid	*n*-hexane fraction of extract from cultivated bulbs	Bulbs	[29]
27	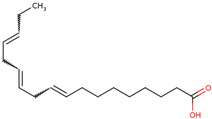 Linolenic acid	4.22	Fatty acid	*n*-hexane fraction of extract from cultivated bulbs	Bulbs	[29]
28	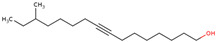 (R)-(−)-14-Methyl-8-hexadecyn-1-ol	0.50	Other	*n*-hexane fraction of extract from cultivated bulbs	Bulbs	[29]
29	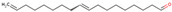 9,17-Octadecadienal, (Z)-	0.10	Other	*n*-hexane fraction of extract from cultivated bulbs	Bulbs	[29]
30	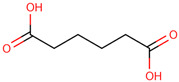 Adipic acid	1.5	Other	*n*-hexane fraction of extract from wild bulbs	Bulbs	[29]
31	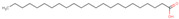 Tricosanoic acid	0.03	Other	*n*-hexane fraction of extract from wild bulbs	Bulbs	[29]
32	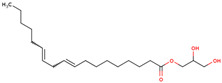 β-Monolinolein	2.30	Other	*n*-hexane fraction of extract from cultivated bulbs	Bulbs	[29]
33	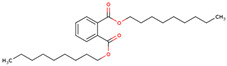 1,2-Benzenedicarboxylic acid, dinonyl ester	5.62	Phthalic acid monoester	*n*-hexane fraction of extract from wild bulbs	Bulbs	[29]
34	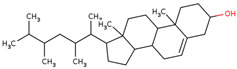 (22R, 24S)-22,24-Dimethylcholesterol	0.50	Sterol	*n*-hexane fraction of extract from cultivated bulbs	Bulbs	[29]
35	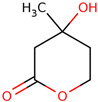 Mevalonic acid lactone	3.7	Other	Dichloromethane fractions	Leaves	[31]
36	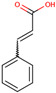 Cinnamic acid	Trace	Carboxylic acid	Dichloromethane fractions	Inflorescences	[31]
37	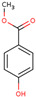 Benzoic acid, 4-hydroxy-, methyl ester	2.2	Other	Dichloromethane fractions	Leaves	[31]
38	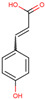 p-Hydroxycinnamic acid	0.9	Phenolic acid	Dichloromethane fractions	Leaves	[31]
39	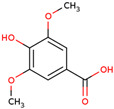 Syringic acid	1.9	Phenolic acid	Dichloromethane fractions	Inflorescences	[31]
40	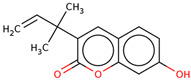 7-Hydroxy-3-(1,1-dimethylprop-2-enyl)coumarin	0.2	Other	Dichloromethane fractions	Leaves	[31]
41	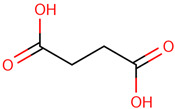 Butanedioic acid	TA	Carboxylic acid	*n-*hexane	Inflorescences	[31]
42	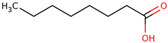 Caprylic acid	0.3	Carboxylic acid	*n-*hexane	Leaves	[31]
43	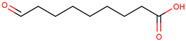 Azelaaldehydic acid	0.6	Carboxylic acid	*n-*hexane	Leaves	[31]
44	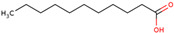 Undecanoic acid	0.1	Carboxylic acid	*n-*hexane	Leaves	[31]
45	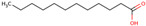 Lauric acid	0.5	Fatty acid	*n-*hexane	Leaves	[31]
46	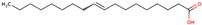 Oleic acid	0.1	Fatty acid	*n-*hexane	Inflorescences	[31]
47	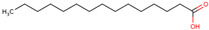 Pentadecanoic acid	0.5	Fatty acid	*n-*hexane	Leaves	[31]
48	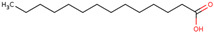 Tetradecanoic acid	0.2	Fatty acid	*n-*hexane	Leaves	[31]
49	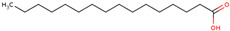 Palmitic acid	19.8	Fatty acid	*n-*hexane	Leaves	[31]
50	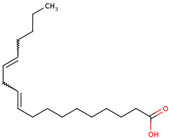 10,13-Octadecadienoic acid	0.2	Fatty acid	*n-*hexane	Leaves	[31]
51	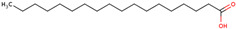 Stearic acid	7.9	Fatty acid	*n-*hexane	Inflorescences	[31]
52	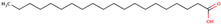 Eicosanoic acid	1.2	Fatty acid	*n-*hexane	Inflorescences	[31]
53	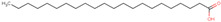 Behenic acid	0.9	Fatty acid	*n-*hexane	Leaves	[31]
54	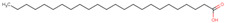 Lignoceric acid	0.6	Fatty acid	*n-*hexane	Inflorescences	[31]
55	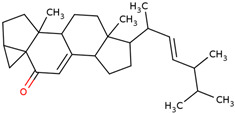 3α, 5-cyclo-ergosta-7,22-dien-6-one	0.3	Phytosterols	*n-*hexane	Inflorescences	[31]
56	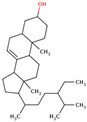 Stigmast-7-en-3-ol, (3β, 5α)-	0.3	Phytosterols	*n-*hexane	Leaves	[31]
57	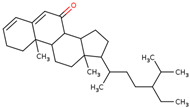 Stigmasta-3,5-dien-7-one	0.3	Phytosterols	*n-*hexane	Inflorescences	[31]
58	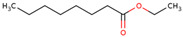 Ethyl caprylate	0.9%^a^		*n-*hexane	Bulbs	[27]
59	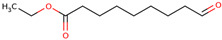 9-oxo-Nonanoic acid, ethyl ester	2.7% ^a^		*n-*hexane	Bulbs	[27]
60	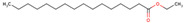 Ethyl palmitate	19.7% ^a^		*n-*hexane	Bulbs	[27]
61	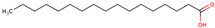 Heptadecanoic acid	2.8% ^a^	Fatty acid	*n-*hexane	Bulbs	[27]
62	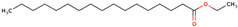 Ethyl heptadecanoate	1.4% ^a^		*n-*hexane	Bulbs	[27]
63	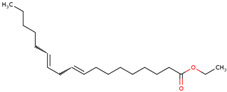 Ethyl linoleate	4.9% ^a^		*n-*hexane	Bulbs	[27]
64	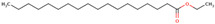 Ethyl stearate	1.5% ^a^		*n-*hexane	Bulbs	[27]
65	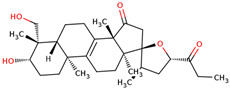 Eucosterol	NP	Phytosterols	Acetone	Bulbs	[15]
66	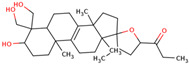 Ketotriol	NP	Phytosterols	Acetone	Bulbs	[17]
67	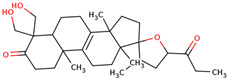 Diketotriol	NP	Phytosterols	Acetone	Bulbs	[17]
68	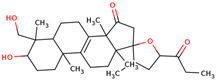 (23*R*)-17,23-epoxy-33,31-dihydroxy-27-nor-5α-lanost-8-ene-15,24-dione	NP	Phytosterols	Acetone	Bulbs	[18]
69	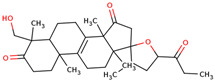 (23*R*)-17,23-epoxy-31-hydroxy-2 7-nor-5α-lanost-8-ene-3,15,24-trione	NP	Other	Acetone	Bulbs	[18]
70	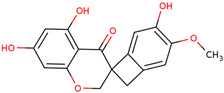 Muscomosin	NP	Homoisoflavonoids	Ether	Bulbs	[25]
71	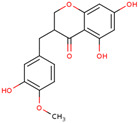 3′-hydroxy-3,9dihydroeucomin	NP	Homoisoflavonoids	Ether	Bulbs	[25]
72	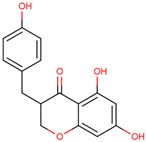 4′demethyl-3,9-dihydroeucomin	NP	Homoisoflavonoids	Ether	Bulbs	[25]
73	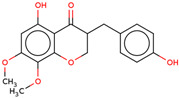 7-*O*-methyl-3,9_dihydropunctatin	NP	Homoisoflavonoids	Ether	Bulbs	[24]
74	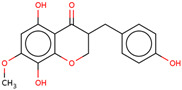 8-*O*-demethyl-7-*O*-methyl-3,9_dihydropunctatin	NP	Homoisoflavonoids	Ether	Bulbs	[24]
75	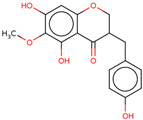 3,9-dihydroeucomnalin	NP	Homoisoflavonoids	Ether	Bulbs	[24]
76	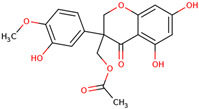 Comosin	NP	Homoisoflavonoids	Ether	Bulbs	[3]
77	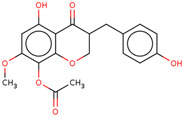 8-*O*-demethyl-8-*O*-acetyl-7-*O*-methyl-3,9dihydropunctatin	NP	Homoisoflavonoids	Ether	Bulbs	[3]
78	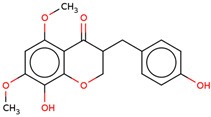 H2	NP	Homoisoflavonoids	Ether	Bulbs	[34]
79	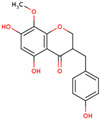 H5	NP	Homoisoflavonoids	Ether	Bulbs	[34]
80	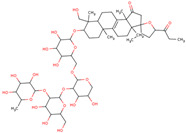 Muscaroside A	NP	Glycoside	Acetone	Bulbs	[20]
81	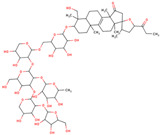 Muscaroside B	NP	Glycoside	Acetone	Bulbs	[21]
82	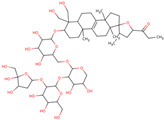 Muscaroside C	NP	Glycoside	Acetone	Bulbs	[22]
83	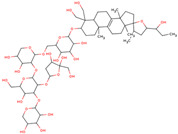 Muscaroside D	NP	Glycoside	Acetone	Bulbs	[23]
84	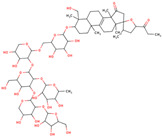 Muscaroside E	NP	Glycoside	Acetone	Bulbs	[23]
85	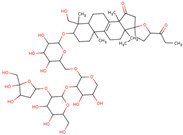 Muscaroside F	NP	Glycoside	Acetone	Bulbs	[23]

^a^ Peak area relative to total peak area percentage (relative area percentage); NP: not provided.

## 6. Bioactivities

The early pharmacological activity of plants is known from its traditional use in society [1]. The different bioactivity studied for LC has primarily been focused on the bulbs of plants, as they have been used as a vegetable since ancient times. However, some activities have also been studied in other aerial parts of the plant such as leaves and inflorescence [31]. The important biological activities of LC plants are reported in the literature, including antioxidant, anti-obesity, anti-cancer, anti-AD, anti-inflammatory, antibacterial, and immune enhancement activities. Among these, the antioxidant activity of the plant has the potential to contribute to other important pharmacological activities such as anti-obesity, anti-cancer, anti-inflammatory, and anti-diabetes [9,35,36,37]. Hence, the antioxidant activity of plants has been analyzed in numerous experimental studies.

### 6.1. Antioxidant Activity

Natural antioxidants are considered a potential pharmacological intervention against deadly diseases and disorders including cancer and neurodegenerative disorders [38,39,40]. Consequently, the research on natural antioxidants for their health benefits has been increasing. Initially, the antioxidant activity of LC was premeditated in a study conducted to evaluate the antioxidant properties of non-cultivated vegetables from the southern part of Italy [5]. The antioxidant potential of all selected vegetable plants was screened through the 1,1-diphenyl-2-picrylhydrazil (DPPH) radical screening assay, which is based on free radical scavenging activity. The extract from the LC bulbs had the highest antioxidant activity among all 27 selected extracts and even more than the reference extract from *Rhodiola rosea* used in the study. Further antioxidant activity of the LC bulb extract was found to be highest in the in vitro non-enzymatic inhibition of the lipid peroxidation in liposomes, which is equivalent to the reference compound quercetin. Similarly, the effective antioxidant activity of LC in xanthine oxidase (XO) inhibition was also observed [5].

Later, metal chelating activity through Fe2+ chelating activity assay, and antioxidant activity through DPPH, ferric-reducing ability power (FRAP), and 2,2′-azino-bis (3-ethylbenzothiazoline-6-sulfonic acid) (ABTS) assays of LC bulb extract were carried out in ethanolic and *n-*hexane fractions. In the FRAP assay, the antioxidant activity of LCB extract in the ethanolic fraction was comparable with the positive control (butyl hydroxytoluene) used in the study [27]. ABTS and DPPH assay again revealed that both extract fractions have reducing power (Table 2).

In a further study, the antioxidant activity of LC bulb extracts extracted through different solvents including ethanol and *n-*hexane was evaluated [28]. The antioxidant activity of the ethanol extract activity was slightly higher than that of the *n-*hexane extract (Table 2).

Similarly, the antioxidant activity of LC bulb extract from wild and cultivated LC was examined through the DPPH assay and β-carotene bleaching test. The antioxidant activity of wild plant bulbs was higher than the cultivated plant bulb in the DPPH assay. Further, the highest antioxidant activity was achieved by the dichloromethane fraction from the bulbs of the cultivated plants. Similar results were obtained in the β-carotene bleaching test: the antioxidant activity of the raw extract from bulbs of wild-grown plants was higher than that from the cultivated plants. The highest antioxidant activity in the β-carotene bleaching test was achieved by ethyl acetate fraction from the bulbs of the cultivated plants [29].

In another study, the antioxidant activities of LC bulbs grown in southern Italy were studied through DPPH, nitric oxide, and superoxide radicals scavenging assays. The strong superoxide anion scavenging activity of LC bulb extract was calculated and found to be better than the positive control (ascorbic acid) used in the study [30]. Similarly, a dose-dependent nitric oxide radical scavenging activity was observed but it was lesser than the positive control [30]. The antioxidant activity in DPPH assays was also identified and it was found to be similar to that reported in the previous studies [27,28].

Considering the dietary aspect of LC bulbs, the extracts from the traditional boiled and steam-cooked bulbs were also studied for their antioxidant properties in the study [12]. In both of the studied methods (i.e., DPPH assay and β-carotene bleaching test) for antioxidant activity, the raw extract was found to have the highest antioxidant activities, which might suggest a small loss of antioxidant activity during the cooking process. In other studies, researchers analyzed the antioxidant activity of extract from the bulbs of LC before animal studies for the anti-obesity properties. In this study, strong antioxidant activity of extract from the bulbs of LC was observed in both experiments, i.e., the DPPH assay and the β-carotene bleaching test [13] (Table 2).

**Figure 2:**
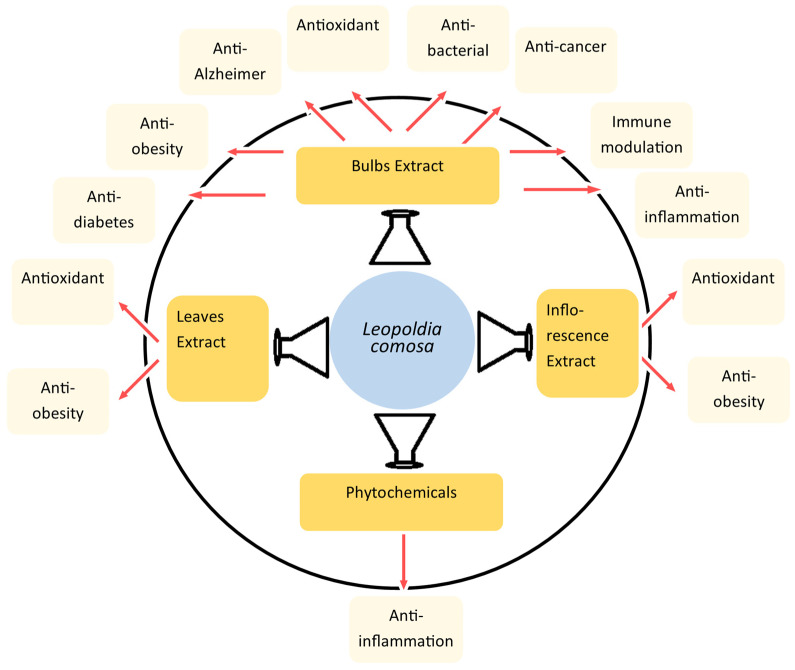
Pharmacological activities of different parts and forms of LC reported in literature.

Later, researchers studied the antioxidant properties of the extract from the different aerial parts of LC, which included leaves and inflorescences [31]. In this study, the highest antioxidant activity was found in leaves as compared to inflorescences in both the studied antioxidant assays, i.e., the DPPH assay and the β-carotene bleaching test (Table 2 and Figure 2). Among the fractions and the raw extract, the antioxidant activity was maximum in ethyl acetate fractions in the case of both leaves and inflorescences [31].

Recently, the antioxidant activities of aqueous (decocted, infused, and macerated) and organic (ethanolic, macerated ethanolic, acetone, macerated acetone, diethyl ether, and macerated diethyl ether) extracts through different polarities have been studied for their antioxidant activities through five different methods, i.e., hydrogen peroxide scavenging assay (HPSA), Trolox equivalent antioxidant capacity, ferric-reducing antioxidant power assay (FRAP), reducing power assay, and DPPH [32]. In HPSA, similar antioxidant activities were observed for all three types of aqueous extracts, but the maximum was in the case of the decocted extract. Among organic extracts, the highest activities were observed for hot diethyl ether extract in all five different methods used in the studies (Table 2).

The antioxidant activity of bulbs extracted through aqueous and other concentrations of methanol has been evaluated with in vitro assays and cell line experiments. Antioxidant assays used in the study include DPPH, total reducing power, nitric oxide, and superoxide radicals scavenging properties. In the cell line study, the antioxidant activities of LC bulb extracts were evaluated by measuring intracellular reactive oxygen species stimulated through tert-butyl hydroperoxide (t-BOOH) and expression of antioxidant-related genes in the HepG2 cells. Pretreatment with extract at low doses decreased the production of ROS, which had been enhanced due to the t-BOOH treatment in the HepG2 cells [33]. Additionally, expression of several genes/markers related with antioxidant activities, such as nuclear factor-erythroid 2 p45-related factor 2 (*NRF2*), superoxide dismutase (*SOD-2*), glutathione peroxidase (*GPX1*), catalase (*CAT*), NADPH quinone oxidase-1 (*NQO1*), ATP binding cassette subfamily C member (*ABCC6*), and ATP binding cassette subfamily G member (*ABCG2*) were studied through RT-PCR experiments. Aqueous and 50% methanolic extracts of LC bulb treatment enhanced the expression of NRF2 in HepG2 cells. Similarly, the expression of *SOD2* was enhanced with a high dose of aqueous and 70% and 50% methanolic extracts. The expression of *GPX1* was increased in the treatment with a high dose of aqueous and a low dose of 70% methanolic extracts. The expression of *NQO1* was enhanced with a low dose of 50% methanolic extract, and the expression of *ABCC6* was increased in the treatment with a high dose of aqueous (Table 2).

**Table 2:** Different biological activities of LC.

Activity	Dose	Method	Result	Refs
Antioxidant activity	Extract from bulbs in ethanol	DPPH	>80%	[5]
		Lipid peroxidation in liposome inhibition assay	>85%	
		XO inhibition	>35%	
	Extract from bulbs in ethanol and *n-*hexane	Metal chelating assay	IC_50_ values of 78.8 (ethanol) and 113.6 (*n-*hexane) μg/mL	[27]
		FRAP	66.7 ± 3.1 (ethanol) and 112.4 ± 2.5 (*n-*hexane) μM Fe/g	
		ABTS	1.7 ± 0.7 and 3.8 ± 0.9 TEAC value	
		DPPH of ethanol and *n-*hexane extracts	IC_50_ values of 40.9 (ethanol) and 46.6 (*n-*hexane) μg/mL,	[28]
	Extract from bulbs of wild and cultivated plants (raw, dichloromethane, and ethyl acetate fractions)	DPPH	IC_50_ values of raw, dichloromethane, and ethyl acetate fractions were 152.90, 38.97, and 31.57 μg/mL, respectively, for the bulbs of wild plants; and the IC_50_ values of dichloromethane and ethyl acetate fractions were 11.25 and 53.24 μg/mL, respectively, for the bulbs of cultivated plants.	[29]
		β-carotene-linoleic acid bleaching assay	IC_50_ values of raw, dichloromethane, and ethyl acetate fractions were 24.68, 17.30, and 57.71 μg/mL, respectively, for the bulbs of wild plants; and the IC_50_ values of dichloromethane and ethyl acetate fractions were 8.54 and 4.51 μg/mL, respectively, for the bulbs of cultivated plants.	
	Methanolic (70%) extract from LC bulbs	DPPH, nitric oxide, and superoxide radicals scavenging assay	IC_50_ = 36.73 μg/mL (DPPH) IC_25_ = 144.13 μg/mL (NO) IC_50_ = 54.15 μg/mL (superoxide anion radical)	[30]
	Ethanolic extracts from bulbs from raw, boiled, and steam-cooked bulbs	DPPH	IC_50_ = 1.34 mg/mL (raw) IC_50_ = 3.59 mg/mL (steam-cooked) IC_50_ = 9.63 mg/mL (boiled)	[12]
		β-carotene-linoleic acid bleaching assay	IC_50_ = 9.13 mg/mL (raw) IC_50_ = 17.37 mg/mL (steam-cooked) IC_50_ = 14.81 mg/mL (boiled)	
	Extract from LC bulbs obtained using a dynamic extractor	DPPH	The IC_50_ = 10.2 ± 0.2 μg/mL (*n* = 5).	[13]
		BCB assay (The IC_50_ of antilipoperoxidant activity, measured by BCB assay, for ascorbic acid was 1.50 ± 0.10 μg/mL.)	IC_50_ = 10.80 ± 0.74 μg/mL (30 min) and 81.4 ± 1.28 μg/mL (60 min) (*n* = 5 for each test).	
	Extract of leaves	DPPH	The IC_50_ values of raw, *n-*hexane, dichloromethane, and ethyl acetate fractions were 154.8, >1000, >1000, and 86.09 μg/mL, respectively, for the extract from leaves; and the	[31]
		BCB assay	dichloromethane and ethyl acetate fractions were 44.46, >100, >100, and 23.73 μg/mL, respectively, for the extract from leaves.	
	Extract from inflorescences	DPPH	The IC_50_ values of raw, *n-*hexane, dichloromethane, and ethyl acetate fractions were 316.6, >1000, 472.1, and 102.4 μg/mL, respectively, for the extract from inflorescences.	
		BCB assay	The IC_50_ values of raw, *n-*hexane, dichloromethane, and ethyl acetate fractions were 84.42, >1000, >1000, and 53.35 μg/mL, respectively, for the extract from inflorescences.	
	Aqueous and organic extract from bulbs	HPSA	The percentages of scavenging activities were 62.12 ± 0.2, 61.89 ± 0.3, 61.72 ± 0.1, 61.3 ± 0.16, 61.09 ± 0.05, 61.24 ± 0.2, 60.94 ± 0.2, 62.67 ± 0.06, and 61.91 ± 0.1 for decocted aqueous, infused aqueous, macerated aqueous, ethanolic, macerated ethanolic, acetone, macerated acetone, diethyl ether, and macerated diethyl, respectively.	[32]
		Trolox equivalent antioxidant capacity (μg of Trolox equivalent per mg of dry plant extract)	The Trolox equivalent (μg TE/mgE) antioxidant capacity values were 27.46 ± 0.69, 17.18 ± 0.17, 6.63 ± 0.31, 225.86 ± 1.04, 89.47 ± 0.68, 364.96 ± 0.28, 343.02 ± 1.44, 381.63 ± 0.63, and 360.93 ± 0.25 for decocted aqueous, infused aqueous, macerated aqueous, ethanolic, macerated ethanolic, acetone, macerated acetone, diethyl ether, and macerated diethyl, respectively.	
		DPPH	The IC_50_ values were 1011.33 ± 4.37, 1089.33 ± 0.92, 1140 ± 20.64, 139.4 ± 6.93, 220.5 ± 2.91, 99.76 ± 0.04, 100 ± 0.03, 10.08 ± 0.01, and 10.15 ± 0.04 μg/mL for decocted aqueous, infused aqueous, macerated aqueous, ethanolic, macerated ethanolic, acetone, macerated acetone, diethyl ether, and macerated diethyl, respectively.	
		FRAP assay (μg of Trolox equivalent per mg of dry plant extract)	The Trolox equivalent (μg TE/mgE) antioxidant activity values were 12.9 ± 0.1, 11.16 ± 0.52, 15.27 ± 0.1, 131.55 ± 0.26, 49.24 ± 0.13, 277.74 ± 0.67, 225.77 ± 0.15, 394.77 ± 0.74, 358.77 ± 0.74 for decocted aqueous, infused aqueous, macerated aqueous, ethanolic, macerated ethanolic, acetone, macerated acetone, diethyl ether, and macerated diethyl, respectively.	
		Reducing power assay (μg of ascorbic acid equivalent per mg of dry plant extract)	The Trolox equivalent (μg TE/mgE) antioxidant activity values were 8.36 ± 0.06, 7.91 ± 0.14, 10.68 ± 0.13, 59.40 ± 0.21, 18.86 ± 0.05, 147.39 ± 1.07, 133.32 ± 0.8, 356.7 ± 0.92, and 283.95 ± 0.59 for decocted aqueous, infused aqueous, macerated aqueous, ethanolic, macerated ethanolic, acetone, macerated acetone, diethyl ether, and macerated diethyl, respectively.	
	Aqueous and methanolic (50 and 70%) extracts from bulbs	DPPH	IC_50_ = 38.02 μg/mL (aqueous) IC_50_ = 29.43 μg/mL (50% methanolic) IC_50_ = 24.60 μg/mL (70% methanolic)	[33]
		Nitric oxide scavenging	IC_50_ = 269.21 μg/mL (aqueous) IC_50_ = 168.52 μg/mL (50% methanolic) IC_50_ = 122.94 μg/mL (70% methanolic)	
		Superoxide radicals scavenging	IC_50_ = 51.43 μg/mL (aqueous) IC_50_ = 50.10 μg/mL (50% methanolic) IC_50_ = 36.50 μg/mL (70% methanolic)	
		Reducing power	37.93 (mg GAE/g) (aqueous) 44.51(mg GAE/g) (50% methanolic) 47.52(mg GAE/g) (70% methanolic)	
	1, 5, 10, 50, 100, and 600 µg/mL	In HepG2 cells the intracellular ROS level was measured with DCFH-DA.	Pre-treatment with low doses (100–1 µg/mL) of the extracts for 24 h protected cells from oxidative stress and ROS ↓.	
	Aqueous and methanolic extracts from bulbs	RT-PCR	*NRF2* ↑ (aqueous and 50% methanolic extracts), *SOD-2* ↑ (aqueous and methanolic extracts), *GPX1* ↑ (aqueous and 70% methanolic extracts), *NQO1* ↑ (50% methanolic extract), *ABCC6* ↑ (aqueous extract)	
Anti-obesity	Extracts from bulbs of wild and cultivated plants in raw, dichloromethane, and ethyl acetate fractions	Lipase inhibition assay (orlistat is taken as positive control IC_50_ = 0.018 mg/mL)	The IC_50_ values of raw, dichloromethane, and ethyl acetate fractions were 0.166, 0.290, and 0.153 mg/mL, respectively, from the bulbs of wild plants; and the IC_50_ values of dichloromethane and ethyl acetate fractions were 0.218 and 0.469 mg/mL, respectively.	[29]
	Leaves and inflorescences of wild and cultivated plants in raw, dichloromethane, and ethyl acetate fractions		The IC_50_ values of raw, *n-*hexane, dichloromethane, and ethyl acetate fractions were 3.819, 0.369, 1.409, and 0.336 mg/mL, respectively, for the leaves of wild plants; and the IC_50_ values of raw, *n-*hexane, dichloromethane, and ethyl acetate fractions were 6.561, 0.736, 1.570, and 0.780 mg/mL, respectively, for the inflorescences of wild plants.	[31]
	Ethanolic extract from LC bulbs	Inhibition assays for pancreatic lipase (positive controls, i.e., orlistat IC_50_ values = 0.19 μg/mL)	IC_50_ values of 70.5 ± 0.89 μg/mL for extract and 57.20 ± 0.19 μg/mL for positive control drug	[13]
		Inhibition assays for pancreatic α-amylase (positive controls i.e., acarbose IC_50_ = 36.50 ± 0.32 μg/mL)	IC_50_ values of 46.3 ± 0.23 μg/mL for extract and 36.50 ± 0.32 μg/mL for positive control drug	
	Wistar rats oral administration of 20 or 60 mg/die for 12 weeks	Anthropometric and metabolic variables	Body weight ↓, circumference of waist ↓ perirenal ↓, retroperitoneal ↓, epididymal ↓, and abdominal fat ↓ weight ↓ and CSI ↓ values of heart and liver, ROS ↓	
		Blood biochemical measurements and HOMA-IR index	ROS production ↓, triglycerides ↓, LDL cholesterol ↓, LDL-cholesterol-ox ↓, and total cholesterol ↓; similarly, the level of plasma insulin ↓, basal glycemia level ↓, and HOMA-IR index ↓	
		Tissue histology	In abdominal fat, the areas of adipocytes ↓. In liver samples, the presence of fat vacuoles ↓, the triglyceride content of liver ↓.	
		WB analysis of key enzymes of gluconeogenesis	The expression of PEPCK ↓ and G6Pase ↓	
Anti-diabetes activity	Extract from LC bulbs in ethanol and *n-*hexane	α-amylase inhibition assay (positive controls i.e., acarbose IC_50_ = 50.0 ± 0.9 μg/mL)	IC_50_ = 81.3 ± 2.7 μg/mL (ethanol) IC_50_ = 166.9 ± 3.4 μg/mL (*n-*hexane)	[27]
		α-Glucosidase (positive controls, i.e., acarbose IC_50_ = 35.5 ± 1.2 μg/mL)	IC_50_ = 112.8 ± 3.3 μg/mL (ethanol) IC_50_ = 200.8 ± 2.8 μg/mL (*n-*hexane)	
	Ethanolic extracts from raw, boiled, and steam-cooked bulbs	α-amylase inhibition assay	IC_50_ values of 0.16 ± 0.03, 0.73 ± 0.13, and 0.69 ± 0.02 mg/mL in RB, SB, and BB, respectively	[12]
	Methanolic extract from LC bulbs	α-amylase (positive control acarbose IC_50_ = 47.33 μg/mL)	IC_50_: 75.17 μg/mL	[30]
		α-glucosidase (positive control acarbose IC_50_ = 33.72 μg/mL)	IC_50_: 85.33 μg/mL	
	Aqueous (decocted, infused, and macerated) and organic (ethanolic, macerated ethanolic, acetone, macerated acetone, diethyl ether, and macerated diethyl) extract from bulbs	α-amylase (positive control acarbose IC_50_ = 616.33 μg/mL)	The IC_50_ values were 1200, 2880, 2752, 2264 2384, 2219, 2289, 2512, and 2897 μg/mL for decocted aqueous, infused aqueous, macerated aqueous, ethanolic, macerated ethanolic, acetone, macerated acetone, diethyl ether, and macerated diethyl, respectively.	[32]
		α-glucosidase inhibition assay (positive control acarbose IC_50_ = 195 μg/mL)	The IC_50_ values were 238.5, 258.9, 268.2, 257.9, 162.7, 85.4, 85.9, 136, and 130.8 μg/mL for decocted aqueous, infused aqueous, macerated aqueous, ethanolic, macerated ethanolic, acetone, macerated acetone, diethyl ether, and macerated diethyl, respectively.	
		β-galactosidase inhibition assay (positive control quercetin IC_50_ = 171.16 μg/mL)	The IC_50_ values were 216, 205, 245, 182, 196, 163, 200, 240, and 291 μg/mL for decocted aqueous, infused aqueous, macerated aqueous, ethanolic, macerated ethanolic, acetone, macerated acetone, diethyl ether, and macerated diethyl, respectively.	
Anti-AD	*n-*hexane and ethanolic extract from bulbs	Acetylcholinesterase and butyrylcholinesterase inhibiting activities	IC_50_ = 131 μg/mL (ethanol) AChE IC_50_ = 282.9 μg/mL (ethanol) BChE IC_50_ = 104.9 μg/mL (*n-*hexane) AChE IC_50_ = 128.1 μg/mL (*n-*hexane) BChE.	[28]
	Methanolic extract from LC bulbs	Acetylcholinesterase inhibition assay (positive control, i.e., galantamine IC_50_ = 8.9760.15 μg/mL)	IC_50_ = 107.6465.38 μg/mL	[30]
Anti-cancer	100, 200, and 400 mg/kg 50% ethanol fraction of aqueous bulb extract	Walker-256 (intramuscular) carcinosarcoma	Weight of tumor decreased	[16]
	Ethanolic extracts from raw and cooked bulbs	MTT assay of MCF-7 cell line	IC_50_ = 10.27 μg/mL (ethanol) raw IC_50_ = 669.3 μg/mL (ethanol) cooked	[12]
Anti-inflammation	Five homoisoflavones and fraction at 100 μg/ear	Croton oil-induced mouse ear dermatitis	27–41% ↓ (in inflammation)	[34]
	Methanolic extract from LC bulbs (25, 50, and 75 mg/mL)	MMP-2 and MMP-9 derived from the primary culture of rat astrocytes activated with LPS detected through gelatin gel zymography and 1,10 phenanthroline used as a positive control	MMP-2 ↓ and MMP-9 ↓ (completely inhibited the activity of MMP-9 at 50 mg/mL)	[30]
Antibacterial activity	Aqueous and ethanolic extracts from LC bulbs	Inhibition of biofilm formation through methicillin-resistant *Staphylococcus aureus* studied through staining with crystal violet	IC_50_ = 8 μg/mL (aqueous) raw IC_50_ = 16 μg/mL (ethanol) cooked	[41]
	Extract of LC bulbs in different solvents: ethanolic, macerated ethanolic, acetone, macerated acetone, diethyl ether, and macerated diethyl.	Agar disc diffusion assay measuring the zone of inhibition formed around the discs against selected bacterial species (*Bacillus subtilis*, *Staphylococcus aureus*, *Listeria innocua*, *Pseudomonas aeruginosa*, *Proteus mirabilis*, and *Escherichia coli*).	Inhibition was observed against *Listeria innocua* and *Proteus mirabilis*.	[32]
Immune stimulation	0.1 mL of the alcoholic plant extract in two different concentrations (0.5 mg/fish and 2 mg/fish)	*Sparus aurata* NBT-positive cells count	NBT-positive cells count ↑	[42]
		Specific growth rate	Growth ↑	
		Lysozyme activity of serum samples	Lysozyme activity ↑	
		Total and differential leukocyte count	level of total leukocyte ↑, neutrophils ↑, monocytes ↑, and eosinophils counts ↑	

ABTS: 2,2′-azino-bis(3-ethylbenzothiazoline-6-sulfonic acid); BB: boiled bulbs; BCB: β-carotene-linoleic acid bleaching; DCFH-DA: 2′,7′-dichlorodihydrofluorescein diacetate; DPPH: 1,1-diphenyl-2-picrylhydrazil; ferric-reducing ability power (FRAP); GAE: gallic acid equivalents; HPSA: hydrogen peroxide scavenging assay; LC: leopoldia comosa; LPS: lipopolysaccharide; MTT: 3-(4,5-dimethylthiazol-2-yl)-2,5-diphenyltetrazolium bromide; NBT: nitroblue tetrazolium; RB: raw bulb; ROS: reactive oxygen species; SB: Steamed bulbs; TE: Trolox equivalent; WB, Western blot; XO: Xanthine oxidase; ↑: up-regulation; ↓: down-regulation/inhibition.

### 6.2. Anti-Diabetes

Over 529 million people were found globally to have diabetes in 2021, which indicates that the prevalence of diabetes is increasing worldwide; this poses a substantial challenge to public health [43]. Natural compounds are believed to have the potential to be developed against diabetes [44,45,46]. The anti-diabetes potential of LC bulbs has been studied by analyzing the inhibition of two important enzymatic drug targets of diabetes, i.e., α-glucosidase and α-amylase. Extract from the bulbs of LC was able to effectively inhibit both drug targets, and in the case of both targets, the inhibition was higher for ethanol extract as compared to *n-*hexane [27]. The relatively low activity of the non-polar extract (*n-*hexane) indicated the important role of the polar component of LC bulbs in anti-diabetes activity [27]. The identification of components important for the activity may be helpful in optimizing the anti-diabetes activity of LC bulbs in further studies.

Considering the dietary use of LC, the extracts from the traditional boiled and steam-cooked bulbs have also been studied for their antidiabetic potential [12]. The extracts from traditionally cooked, steam-cooked, and raw bulbs were studied for their inhibitory activity against α-amylase. A higher inhibition activity was observed in the case of the extract from raw bulbs. The significant difference between the properties of the cooked and raw extracts indicated that cooking slightly reduced the α-amylase inhibition activity of the extract from the cooked bulbs (Table 2). Later, the researcher studied the anti-diabetes potential of methanolic extract from LC bulbs along with other biological properties. Anti-diabetes activity of the extract against α-amylase and α-glucosidase was studied using acarbose as a positive control.

In a recent study, the anti-diabetes investigation was conducted with the inclusion of an inhibitory assay of one more important target in diabetes, i.e., β-galactosidase along with α-glucosidase and α-amylase [32]. Different extracts of aqueous (decocted, infused, and macerated) and organic (ethanolic, macerated ethanolic, acetone, macerated acetone, diethyl ether, and macerated diethyl ether) LC bulbs were used to study their activity against all three selected drug targets. Strong inhibition was observed for organic extracts, particularly the acetone extract, against β-galactosidase and α-glucosidase enzymes, which was better than their control compounds (acarbose and quercetin for α-glucosidase and β-galactosidase, respectively) used in the experiments. In the case of α-amylase, the most active extract was decocted aqueous extract, but its activity was lower than the positive control (acarbose) used in the experiments (Table 2).

### 6.3. Anti-Obesity

The worldwide occurrence of overweight and obesity is worryingly growing and also leading to several other serious public health concerns [47]. As with diabetes, the natural compounds are believed to have the potential to be developed against obesity [48,49]. The accumulation of excessive fat and its inappropriate storage is considered as obesity; hence, fat and the dysregulation of its metabolism can be considered as the primary factors responsible for obesity in the population. Pancreatic lipase is the enzyme that helps with the digestion and absorption of fat from food in the intestine. Inhibiting pancreatic lipase is one of the proven strategies for the treatment of obesity. Orlistat is the only conventional drug used for obesity treatment through the inhibition of pancreatic lipase, but the side effects of the drug have ignited the hunt to develop plant-based lipase-inhibiting drugs for obesity [50]. The pancreatic lipase inhibition activity of LC bulb extract from wild and cultivated plants has been studied to explore its anti-obesity potential (Figure 2). Importantly, raw and polar fractions of extract from wild-grown plants showed good lipase-inhibiting activity in the assays, which clearly indicated the anti-obesity potential of LC bulb extract. The study also suggests that the polyphenols, which can be considered bioactive metabolites of the plant, might be responsible for the inhibition of pancreatic lipase activity, but further research may be required to confirm this [29]. Similarly, anti-obesity potential through the inhibition of pancreatic lipase has also been studied for the extract from the leaves and inflorescence of LC [31]. Lipase-inhibition activities were found to be higher in the leaves than in the inflorescence in the raw extract and different fractions used in the study (Table 2). Significant lipase-inhibition activities of both of these aerial parts of LC suggest the anti-obesity potential of these parts along with bulbs. Further studies aimed at the identification of components important for anti-obesity activity would help to optimize and develop LC as an anti-obesity therapeutic [31]. Encouraging results of anti-obesity activities from previous in vitro studies inspired further in vivo anti-obesity studies using a rat model [13,31]. Two different doses of extract from LC bulbs and a positive control drug (orlistat) were used in animal studies in Wistar rats. The doses were selected according to the results from pancreatic lipase and pancreatic α-amylase inhibition assays, conducted before the animal study. In both enzyme inhibition studies, the extract from LC bulbs achieved comparable activities with positive controls, i.e., orlistat and acarbose in the case of pancreatic lipase, and pancreatic α-amylase inhibition assays, respectively. In the animal study, the body weight and circumference of the waist of rats in both treatment groups were lower than in the high-fat diet (HFD) group after 12 weeks of the oral administration of extract from LC bulbs. Additionally, the perirenal, retroperitoneal, epididymal, and abdominal fat in the rats in both treatment groups was lower than in the high-fat diet group after treatment [13]. Similarly, the weight and CSI values of the heart and liver in the high-fat diet group were enhanced, which was then reversed by the treatment with LC bulb extract. In blood, ROS production, triglycerides, LDL cholesterol, LDL-cholesterol-ox, and total cholesterol were increased in the high-fat diet group compared to the control group, while being significantly reduced in both low and high-dose treatment groups. Similarly, the level of plasma insulin, basal glycemia level, and homeostatic model assessment-estimated insulin resistance (HOMA-IR) index of the high-fat diet group were enhanced in comparison with the control group, which was also reversed by the treatment with LC bulb extract in both treatment groups [13].

In tissue histology of abdominal fat, the areas of adipocytes were increased in the HFD group compared to the control group, while being significantly reduced in the high-dose (HFD + Lc (60 mg)) treatment group (Table 2). Similarly, in liver samples, the presence of fat vacuoles was strongly increased in the HFD group compared to the control group, while being significantly reduced in the high-dose (HFD + Lc (60 mg)) treatment group. The triglyceride content of the liver was also increased in the HFD group and significantly reduced in both low- and high-dose treatment groups. Additionally, the expression of key enzymes of gluconeogenesis PEPCK and G6Pase was also found to be increased in the HFD group compared to the control group, while being significantly reduced in the high-dose (HFD + Lc (60 mg)) treatment group. The in vitro antioxidant activity and reduction of antioxidant factors such as ROS and LDL-cholesterol-ox in the high-fat diet animal model observed in the study suggested that the antioxidant property of the extract may be an important factor in the anti-obesity effect in both metabolic and anthropometric observations. It was also suggested that the anti-obesity activity of LC may be due to the antioxidant activity of the extract, as factors such as ROS and LDL-cholesterol-ox were found to be reversed in the HFD animals with extract treatment [13].

### 6.4. Antibacterial

Antimicrobial resistance is one of the current important public health concerns that demands the rapid discovery of new/novel antibacterial drugs [51]. Different plants were selected from southern Italy to study their potential to inhibit growth and biofilm formation against methicillin-resistant *Staphylococcus aureus*. A total of 168 extracts from the plants, including aqueous and ethanolic extracts from LC bulbs, were studied [41]. Both extracts ofromLC bulbs showed strong inhibition of biofilm formation, and LC was among the top 10 biofilm-inhibiting extracts with IC50 ≤ 32 µg/mL.

The antibacterial activity of extract from LC bulbs was carried out against different gram-positive (*Bacillus subtilis*, *Staphylococcus aureus*, and *Listeria innocua*) and negative bacteria (*Pseudomonas aeruginosa*, *Proteus mirabilis*, and *Escherichia coli*) causing common infectious diseases. The agar disc diffusion assay was used to study the inhibition of selected bacterial strains with different doses of extract. The organic extract from LC bulbs showed antibacterial activity against both gram-positive and negative bacteria but it was limited to only two bacterial species selected in the study, i.e., *Proteus mirabilis* and *Listeria innocua*, and it was lower than the positive control used in the study [32]. Importantly, the inhibition of *Listeria innocua* was only observed in the diethyl extracts, which suggests the importance of the components present in these extracts. These initial antibacterial studies highlighted the importance of components imperative for antibacterial activity and the identification of these components may be useful for the optimization of the antibacterial properties of the extract for further development as an antibacterial therapeutic candidate.

### 6.5. Immune Enhancement Effect of LC Bulb Extracts

Several herbal products are considered to have potential immune modulation properties [52,53]. Studies have shown that immune stimulation is not only found to be useful in fighting against infectious diseases but it may also help against other important noninfectious diseases such as cancer [8]. Considering the importance of immune stimulation in aquaculture, the immune-stimulating effect of the extract from LC bulbs was studied in gilt-head seabream, *Sparus aurata* [42]. The extract was investigated at two different doses (0.5 and 2 mg) through intraperitoneal injection, and both doses were found to enhance different parameters associated with immune stimulation. Blood parameters that were found to be increased in the study were nitroblue tetrazolium (NBT)-positive cell count, lysozyme activities of serum samples level of total leukocyte, neutrophils, monocytes, and eosinophils counts [42]. The growth of the fish was also found to be increased after the administration of extracts. Additionally, there was no negative effect of the extract treatment observed on the hematocrit level of blood in the study. The study concluded that the extract from LC bulbs may enhance the nonspecific immune system of *Sparus aurata*, which can be further studied on other fishes in aquaculture.

### 6.6. Anti-Cancer Activity

Like diabetes and obesity, the global burden of cancer is also increasing, rapidly surpassing the control capacity [54]. Natural products are considered important potential anti-cancer agents due to their accessibility, applicability, and reduced cytotoxicity [55]. In the initial study, the anticancer activity of ethanol-precipitated fraction of extract from LC bulbs was studied on Walker 256 (intramuscular) carcinosarcoma [16]. Before this experiment, the toxicity study on the DBA strain of mice was carried out. The daily dose of 2 g/kg was found to be tolerated by all the mice used in the study, and no side effects were observed. It was concluded that the water-soluble part of the extract had less toxicity. Different fractions of the extract were prepared according to the volume of ethanol used for the precipitation. Among these, the most active fraction demonstrated a reduction in tumor volume at 1:5 (treatment/control) compared with control (Table 2) [16].

Later, the anti-tumor activity of LC bulbs was studied on human breast adenocarcinoma-derived MCF-7 cell lines. The effect of the extract on cell viability was studied through a 3-(4,5-dimethylthiazol-2-yl)-2,5-diphenyltetrazolium bromide (MTT) assay. The extracts from the raw and cooked bulbs were examined for anti-proliferative activities. Effective dose-dependent anti-proliferative activity of extract from the raw bulbs was observed in the study [12]. In the case of cooked bulbs, the activity dropped and was only observed at a high dose, which suggested that cooking reduced the anti-proliferative activity of the extract such as antioxidant and anti-diabetes activities, which were reported to be reduced in the cooked bulbs in comparison with raw bulbs. Thus, cooking may reduce the content of biologically active components of the extract.

### 6.7. Anti-Inflammatory

Inflammation may lead to other lethal diseases or conditions including cancer, cardiovascular disease, diabetes, non-alcoholic fatty liver disease, and neurodegenerative and autoimmune disorders [56]. Hence, anti-inflammatory activity can be considered an important activity, as it may be preventive as well as therapeutic in other deadly diseases, including cancer and AD [57,58,59]. In the initial study, the presence of newly discovered homoisoflavanones that were structurally similar to flavonoids was also speculated to have biological activity like flavonoids. Therefore, the researcher studied the anti-inflammatory activity of homoisoflavanones containing a fraction of extract from LC bulbs through croton oil-induced dermatitis in a mouse model [34]. Effective anti-inflammatory activity (comparable with indomethacin-positive control drug) observed for the fraction inspired the further isolation of 5 homoisoflavanones from the fraction for anti-inflammatory activity (Figure 2). The anti-inflammatory activity was observed in all five compounds in a dose-dependent manner, and the inhibition of inflammation ranged from 21 to 41% at 100 mg/ear (Table 2). Among them, 4′demethyl-3,9-dihydroeucomin was the most active and lightweight (lowest molecular weight) homoisoflavanone, which may be further studied for anti-inflammatory activities in in vivo experiments (Table 1).

**Figure 3:**
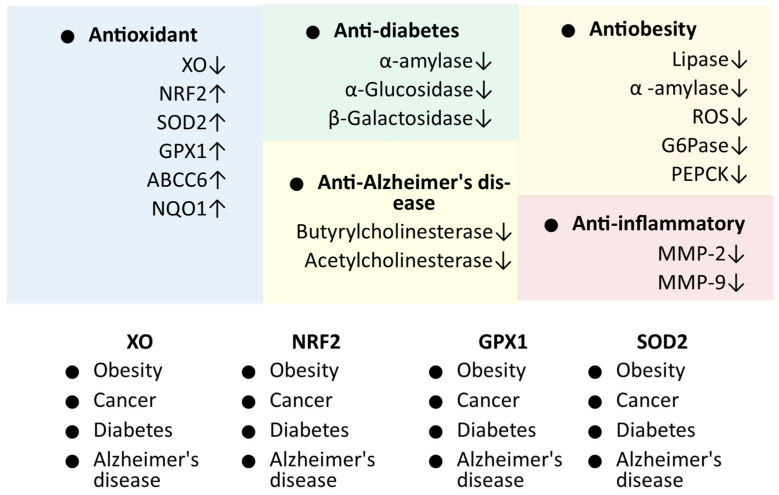
Markers/targets associated with the pharmacological activities of LC. Genes/markers found to be linked with other diseases are listed on the white background. Abbreviations: ↑, up-regulation; ↓, down-regulation/inhibition.

The inflammatory activity of the extract from LC bulbs was also evaluated along with antioxidant and other activities (Figure 2). This activity was studied through the inhibition of MMP-9 and MMP-2 enzymes, which are considered important markers in inflammation response (Figure 3). These enzymes are considered important therapeutic targets, as they are found to be up-regulated in inflammatory conditions. The inhibition of MMP-2 and MMP-9 derived from the primary culture of rat astrocytes activated with lipopolysaccharide (LPS) was examined through gelatin gel zymography [30]. In the study, the strong inhibitory activity of the extract from LC bulbs was able to entirely inhibit MMP-9 at 50 mg/mL concentration. In the case of MMP-2, the same concentration of extract was able to reduce it to 55.664.3% (Table 2).

### 6.8. Anti-Alzheimer’s Disease

Alzheimer’s disease (AD) is the most common neurodegenerative disorder with no cure, and its incidence is alarmingly high in old age. In recent years, several natural compounds have shown strong potential against AD [60,61]. The anti-AD activity of LC bulbs was studied through the inhibitory potential against the two main types of cholinesterase, including acetylcholinesterase and butyrylcholinesterase (Figure 3), which are the known drug targets in AD and other neurodegenerative disorders [62]. The *n-*hexane and ethyl alcohol extracts from LC bulbs inhibited acetylcholinesterase and butyryl cholinesterase enzymes in the study, and *n-*hexane extract had better inhibition in both drug target enzymes, compared to ethyl alcohol extract [28]. Later, in a different study, the methanolic extract from LC bulbs was prepared to study its different biological properties, including anti-AD activity through the inhibition of acetylcholinesterase [30]. Similar to the previous study, the inhibition of acetylcholinesterase in terms of IC_50_ was observed in this study (Table 2). The study suggested that the inhibition of acetylcholinesterase may improve neuromuscular signaling and cognitive functions, which might be helpful in neurodegenerative disorders such as Parkinson’s disease and schizophrenia, along with AD [30].

## 7. Discussion and Future Directions

Along with traditional gastronomy use as a vegetable, LC has shown promising results in several diseases and conditions, which makes it a wonderful nutraceutical candidate that can be developed in further studies. However, different challenges and gaps persist for its different pharmacological properties, which may require systematic and directional research efforts.

Early phytochemical studies of LC were focused on the isolation and identification of individual or several compounds from the extract [15,20,21,22,23]. In later studies, the quantification of the whole class of phytochemicals such as phenolic acid, tannins, and flavonoids was conducted [27,28,29,31]. In most of these studies, the profiling of several phytochemicals present in the LC extracts was also conducted, which resulted in the identification and quantification of possible phytochemicals in these extracts [29,31]. In recent studies, the optimal extract types and conditions were studied to maximize the concentration of phytochemicals responsible for the reported and possible biological activities. The antioxidant properties of LC were found to be highest in comparison with reference and other 27 extracts of different plants [5], and they can be considered among the most prospective properties, as they can support other important properties of LC such as anticancer, anti-obesity, and antidiabetic [9]. Furthermore, the antioxidant properties of LC were strongly validated through various antioxidant assays and methods including DPPS, ABTS, lipid peroxidation, metal chelating, FRAP, HPSA, superoxide radicals, and NO scavenging assays (Table 2). Additionally, LC exerted an antioxidant effect through the enhanced expression of *NRF*, *SOD*, and glutathione peroxidase in cell line studies, and the reduction of ROS in the cell line and blood of rat models used in the anti-obesity study [13]. Importantly, most of the genes (*Nrf2* [63,64,65,66], XO [67,68,69,70], *GpX1* [7,70,71,72], and *SOD2* [6,70,73,74]) targeted through LC for antioxidant activity are also associated with all other diseases studied for LC impact, i.e., diabetes, cancer, AD, and obesity (Figure 3). Studies to validate these targets strongly suggested that it would not only establish the antioxidant potential of LC but also the development of LC against these diseases. However, in numerous studies, the excellent antioxidant activity of LC was established through cell culture and in vitro experiments. Still, an effective study through in vivo experiments for the antioxidant effect of LC is missing in the scientific literature. An analysis of the antioxidant activity of LC in different organs in animal studies is suggested, which might be helpful to utilize the optimal antioxidant potential of LC, especially in the brain.

Similarly, the anti-diabetes activity of LC has not yet been conducted with an animal model, which is strongly suggested for future studies. Furthermore, antidiabetic activity was observed by inhibiting important target enzymes including α-amylase, α-glucosidase, and β-galactosidase. The maximum inhibition activities of α-glucosidase and β-galactosidase were better than the control compounds in organic extract, but the maximum inhibition in the case of α-amylase was observed in aqueous extract [32]. The difference in the inhibition effects of extracts on different target enzymes indicated that the different phytochemicals may be responsible for the anti-diabetes effect of LC. These results emphasize the importance of knowledge of the phytochemical profile of the extracts, as they can be responsible for anti-diabetes activity in LC and its development as a candidate against diabetes. Hence, the identification of phytochemicals present in the (aqueous and organic) extracts is proposed for further studies.

Similarly, the effective inhibition of pancreatic lipase was observed through in vitro experiments, which supported the anti-obesity effects of LC, also confirmed through animal studies. Further clinical studies may be suggested for LC after the safety studies with other animal models. The study also suggested that polyphenol present in the plant may be responsible for the anti-obesity activity of the plant, but further studies would be required to confirm and identify the phytochemicals responsible for it.

Like antioxidant activity, immune modulation can also be considered as a prospective property of LC, as it can help to fight against various infectious and important non-infectious diseases, including cancer [8]. So far, the immune modulation activity of CL has been observed in fish only; therefore, further studies for immune modulation must be conducted on other animal models before its further consideration in clinical studies.

The anti-AD potential of LC is also in its early phase. However, in different studies, the anti-AD potential of extracts from the bulbs of LC was observed against two important target enzymes of AD, i.e., acetylcholinesterase and butyrylcholinesterase. These targeted enzymes are also important in other neurological disorders, including PD and schizophrenia, which suggests possible positive outcomes of LC use in other neurodegenerative disorders. However, neurodegenerative disorders are complex diseases of the brain, and crossing the blood–brain barrier may also be the decisive factor for candidate drugs against AD. Hence, it is suggested that anti-AD activity is studied in further animal models, along with pharmacokinetics and bioavailability studies.

Anti-cancer activities of LC extracts were observed in both in vitro cell lines and animal experiments focusing on different cancers, which creates the possibility of LC being used against other important cancers such as lung and gastric cancer. However, more studies are required to establish the anti-cancer properties of LC in other cancer cell lines and animal models. To optimize the anti-cancer activity, further exploration of phytochemical constituents of the extracts, responsible for the anticancer activity, needs to be conducted. No effective mechanistic study to identify the action mechanism of anti-cancer activities of LC is available. Hence, it is proposed to conduct studies to identify the action mechanism behind the anti-cancer activity of LC in further studies, which would be helpful in developing LC as an intervention against cancer.

Like the limited use of LC bulbs as vegetables, their pharmacological potential is also underutilized. The compiled information on phytochemicals and pharmacological activities, along with highlighted gaps and suggested precise directions, may be helpful in the development of LC as a therapeutic or supplement against the above-discussed important diseases or conditions. The high mineral content and presence of health-promoting phytochemicals highlighted in the current review may also encourage the use of LC as a vegetable.

## Data Availability

Data are contained within this article.

## References

[1] Casoria P., Menale B., Muoio R., Botanico O. (1999). *Muscari comosum*, Liliaceae, in the food habits of south Italy. Econ. Bot..

[2] Boulfia M., Lamchouri F., Lachkar N., Khabbach A., Zalaghi A., Toufik H. (2021). Socio-economic value and ethnobotanical study of Moroccan wild plant: *Leopoldia comosa* L. bulbs. Ethnobot. Res. Appl..

[3] Adinolfi M., Barone G., Belardini M., Lanzetta R., Laonigro G., Parrilli M. (1985). Homoisoflavanones from *Muscari comosum* bulbs. Phytochemistry.

[4] Motti R., Antignani V., Idolo M. (2009). Traditional plant use in the Phlegraean fields Regional Park (Campania, Southern Italy). Hum. Ecol..

[5] Pieroni A., Janiak V., Dürr C., Lüdeke S., Trachsel E., Heinrich M. (2002). In vitro antioxidant activity of non-cultivated vegetables of ethnic Albanians in southern Italy. Phytother. Res. Int. J. Devoted Pharmacol. Toxicol. Eval. Nat. Prod. Deriv..

[6] Liu Y., Qi W., Richardson A., Van Remmen H., Ikeno Y., Salmon A.B. (2013). Oxidative damage associated with obesity is prevented by overexpression of CuZn-or Mn-superoxide dismutase. Biochem. Biophys. Res. Commun..

[7] Zhao Y., Wang H., Zhou J., Shao Q. (2022). Glutathione peroxidase GPX1 and its dichotomous roles in cancer. Cancers.

[8] Jaiswal V., Cho Y.-I., Lee H.-J. (2021). Preliminary Study to Explore the Immune-Enhancement Mechanism of Platycodon grandiflorus Extract through Comparative Transcriptome Analysis. Appl. Sci..

[9] Jaiswal V., Lee H.-J. (2022). Antioxidant activity of Urtica dioica: An important property contributing to multiple biological activities. Antioxidants.

[10] Mulholland D.A., Schwikkard S.L., Crouch N.R. (2013). The chemistry and biological activity of the Hyacinthaceae. Nat. Prod. Rep..

[11] Canale A., Benelli G., Benvenuti S. (2014). First record of insect pollinators visiting *Muscari comosum* (L.) Miller (*Liliaceae-Hyacinthaceae*), an ancient Mediterranean food plant. Plant Biosyst.-Int. J. Deal. All Asp. Plant Biol..

[12] Casacchia T., Sofo A., Casaburi I., Marrelli M., Conforti F., Statti G.A. (2017). Antioxidant, enzyme-inhibitory and antitumor activity of the wild dietary plant *Muscari comosum* (L.) Mill. Int. J. Plant Biol..

[13] Casacchia T., Scavello F., Rocca C., Granieri M., Beretta G., Amelio D., Gelmini F., Spena A., Mazza R., Toma C. (2019). *Leopoldia comosa* prevents metabolic disorders in rats with high-fat diet-induced obesity. Eur. J. Nutr..

[14] Foti C., Nettis E., Cassano N., Damiani E., Ferrannini A., Vena G. (2007). Allergy to *Muscari comosum* bulb. Allergy.

[15] Parrilli M., Lanzetta R., Dovinola V., Adinolfi M., Mangoni L. (1981). Glycosides from *Muscari comosum*. 1. Eucosterol glycoside and structure of its methanolysis products. Can. J. Chem..

[16] Prescott B., Caldes G. (1968). Chemical studies of an antitumor substance from African onions. Proc. Soc. Exp. Biol. Med..

[17] Parrilli M., Lanzetta R., Adinolfi M., Mangoni L. (1980). Glycosides from m uscari comosum—III: The structure of further authentic aglycones. Tetrahedron.

[18] Adinolfi M., Barone G., Lanzetta R., Laonigro G., Mangoni L., Parrilli M. (1984). Triterpenes from Bulbs of *Muscari comosum*, 3. The Structure of Two Minor Novel Nortriterpene Components. J. Nat. Prod..

[19] Adinolfi M., Barone G., Lanzetta R., Laonigro G., Mangoni L., Parrilli M. (1984). Triterpenes from bulbs of *Muscari comosum*, 4. The structure of further novel nortriterpene components. J. Nat. Prod..

[20] Adinolfi M., Barone G., Lanzetta R., Laonigro G., Mangoni L., Parrilli M. (1983). Glycosides from *Muscari comosum*. 4. Structure of muscaroside A. Can. J. Chem..

[21] Adinolfi M., Barone G., Lanzetta R., Laonigro G., Mangoni L., Parrilli M. (1984). Glycosides from *Muscari comosum*. 5. structure of muscaroside B. Can. J. Chem..

[22] Lanzetta R., Laonigro G., Parrilli M., Breitmaier E. (1984). Glycosides from *Muscari comosum*. 6. Use of homo-and heteronuclear two-dimensional nuclear magnetic resonance spectroscopy for the structure determination of the novel glycoside muscaroside C. Can. J. Chem..

[23] Adinolfi M., Barone G., Corsaro M.M., Lanzetta R., Mangoni L., Parrilli M. (1987). Glycosides from *Muscari comosum*. 7. Structure of three novel muscarosides. Can. J. Chem..

[24] Adinolfi M., Barone G., Belardini M., Lanzetta R., Laonigro G., Parrilli M. (1984). 3-Benzyl-4-chromanones from *Muscari comosum*. Phytochemistry.

[25] Adinolfi M., Barone G., Lanzetta R., Laonigro G., Mangoni L., Parrilli M. (1985). Three 3-benzyl-4-chromanones from *Muscari comosum*. Phytochemistry.

[26] Borgonovo G., Caimi S., Morini G., Scaglioni L., Bassoli A. (2008). Taste-active compounds in a traditional Italian food: ‘Lampascioni’. Chem. Biodivers..

[27] Loizzo M.R., Tundis R., Menichini F., Pugliese A., Bonesi M., Solimene U., Menichini F. (2010). Chelating, antioxidant and hypoglycaemic potential of *Muscari comosum* (L.) Mill. bulb extracts. Int. J. Food Sci. Nutr..

[28] Loizzo M.R., Tundis R., Menichini F., Bonesi M., Frega N., Menichini F. (2011). Radical scavenging activity and cholinesterase inhibitory activity of *Leopoldia comosa* (L.) bulbs. Prog. Nutr..

[29] Marrelli M., La Grotteria S., Araniti F., Conforti F. (2017). Investigation of the Potential Health Benefits as Lipase Inhibitor and Antioxidant of *Leopoldia comosa* (L.) Parl.: Variability of Chemical Composition of Wild and Cultivated Bulbs. Plant Foods Hum. Nutr..

[30] Larocca M., Di Marsico M., Riccio P., Rossano R. (2018). The in vitro antioxidant properties of *Muscari comosum* bulbs and their inhibitory activity on enzymes involved in inflammation, post-prandial hyperglycemia, and cognitive/neuromuscular functions. J. Food Biochem..

[31] Marrelli M., Araniti F., Statti G., Conforti F. (2019). Metabolite profiling and biological properties of aerial parts from *Leopoldia comosa* (L.) Parl.: Antioxidant and anti-obesity potential. South Afr. J. Bot..

[32] Boulfia M., Lamchouri F., Senhaji S., Lachkar N., Bouabid K., Toufik H. (2021). Mineral content, chemical analysis, in vitro antidiabetic and antioxidant activities, and antibacterial power of aqueous and organic extracts of Moroccan *Leopoldia comosa* (L.) parl. Bulbs. Evid.-Based Complement. Altern. Med..

[33] Giglio F., Castiglione Morelli M.A., Matera I., Sinisgalli C., Rossano R., Ostuni A. (2021). *Muscari comosum* L. Bulb extracts modulate oxidative stress and redox signaling in hepg2 cells. Molecules.

[34] Loggia R.D., Del Negro P., Tubaro A., Barone G., Parrilli M. (1989). Homoisoflavanones as Anti-inflammatory Principles of *Muscari comosum*. Planta Med..

[35] Jaiswal V., Park M., Lee H.-J. (2021). Comparative transcriptome analysis of the expression of antioxidant and immunity genes in the spleen of a Cyanidin 3-O-Glucoside-treated alzheimer’s mouse model. Antioxidants.

[36] Kumari B., Chauhan K., Trivedi J., Jaiswal V., Kanwar S.S., Pokharel Y.R. (2018). Benzothiazole-Based-Bioconjugates with Improved Antimicrobial, Anticancer and Antioxidant Potential. ChemistrySelect.

[37] Jaiswal V., Chauhan S., Lee H.-J. (2021). The bioactivity and phytochemicals of *Pachyrhizus erosus* (L.) Urb.: A multifunctional underutilized crop plant. Antioxidants.

[38] Marino A., Battaglini M., Moles N., Ciofani G. (2022). Natural Antioxidant Compounds as Potential Pharmaceutical Tools against Neurodegenerative Diseases. ACS Omega.

[39] Marino P., Pepe G., Basilicata M.G., Vestuto V., Marzocco S., Autore G., Procino A., Gomez-Monterrey I.M., Manfra M., Campiglia P. (2023). Potential Role of Natural Antioxidant Products in Oncological Diseases. Antioxidants.

[40] Park D.H., Park J.Y., Kang K.S., Hwang G.S. (2021). Neuroprotective Effect of Gallocatechin Gallate on Glutamate-Induced Oxidative Stress in Hippocampal HT22 Cells. Molecules.

[41] Quave C.L., Plano L.R., Pantuso T., Bennett B.C. (2008). Effects of extracts from Italian medicinal plants on planktonic growth, biofilm formation and adherence of methicillin-resistant Staphylococcus aureus. J. Ethnopharmacol..

[42] Baba E., Uluköy G., Mammadov R. (2014). Effects of *Muscari comosum* extract on nonspecific immune parameters in gilthead seabream, *Sparus aurata* (L. 1758). J. World Aquac. Soc..

[43] Ong K.L., Stafford L.K., McLaughlin S.A., Boyko E.J., Vollset S.E., Smith A.E., Dalton B.E., Duprey J., Cruz J.A., Hagins H. (2023). Global, regional, and national burden of diabetes from 1990 to 2021, with projections of prevalence to 2050: A systematic analysis for the Global Burden of Disease Study 2021. Lancet.

[44] Choudhury H., Pandey M., Hua C.K., Mun C.S., Jing J.K., Kong L., Ern L.Y., Ashraf N.A., Kit S.W., Yee T.S. (2018). An update on natural compounds in the remedy of diabetes mellitus: A systematic review. J. Tradit. Complement. Med..

[45] Lee D., Park J.Y., Lee S., Kang K.S. (2021). In Vitro Studies to Assess the α-Glucosidase Inhibitory Activity and Insulin Secretion Effect of Isorhamnetin 3-O-Glucoside and Quercetin 3-O-Glucoside Isolated from Salicornia herbacea. Processes.

[46] Kim H.-S., Lee D., Seo Y.-H., Ryu S.-M., Lee A.-Y., Moon B.-C., Kim W.-J., Kang K.-S., Lee J. (2023). Chemical Constituents from the Roots of *Angelica reflexa* That Improve Glucose-Stimulated Insulin Secretion by Regulating Pancreatic β-Cell Metabolism. Pharmaceutics.

[47] Tsur A.M., Twig G. (2022). The actual burden of obesity—Accounting for multimorbidity. Lancet Diabetes Endocrinol..

[48] Kang N., Oh S., Kim S.-Y., Ahn H., Son M., Heo S.-J., Byun K., Jeon Y.-J. (2022). Anti-obesity effects of Ishophloroglucin A from the brown seaweed *Ishige okamurae* (Yendo) via regulation of leptin signal in ob/ob mice. Algal Res..

[49] Lee J.H., Lee S., Park J.Y., Park I.-H., Kang K.S., Shin M.-S. (2023). The Beneficial Effect of Salicornia herbacea Extract and Isorhamnetin-3-O-glucoside on Obesity. Processes.

[50] Rajan L., Palaniswamy D., Mohankumar S.K. (2020). Targeting obesity with plant-derived pancreatic lipase inhibitors: A comprehensive review. Pharmacol. Res..

[51] Tang K.W.K., Millar B.C., Moore J.E. (2023). Antimicrobial resistance (AMR). Br. J. Biomed. Sci..

[52] Min S.J., Kim S.J., Park J.Y., Seo C.-S., Choi Y.-K. (2023). Preparation of Herbal Extracts for Intestinal Immune Modulation Activity Based on In Vitro Screening and In Vivo Evaluation of *Zingiber officinale* Rosc. Extracts. Mol..

[53] Gasmi A., Shanaida M., Oleshchuk O., Semenova Y., Mujawdiya P.K., Ivankiv Y., Pokryshko O., Noor S., Piscopo S., Adamiv S. (2023). Natural Ingredients to Improve Immunity. Pharmaceuticals.

[54] Sarfati D., Gurney J. (2022). Preventing cancer: The only way forward. Lancet.

[55] Hashem S., Ali T.A., Akhtar S., Nisar S., Sageena G., Ali S., Al-Mannai S., Therachiyil L., Mir R., Elfaki I. (2022). Targeting cancer signaling pathways by natural products: Exploring promising anti-cancer agents. Biomed. Pharmacother..

[56] Furman D., Campisi J., Verdin E., Carrera-Bastos P., Targ S., Franceschi C., Ferrucci L., Gilroy D.W., Fasano A., Miller G.W. (2019). Chronic inflammation in the etiology of disease across the life span. Nat. Med..

[57] Jaiswal V., Lee H.-J. (2024). Pharmacological Properties of Shionone: Potential Anti-Inflammatory Phytochemical against Different Diseases. Molecules.

[58] Oh H.S., Seo H.J. (2023). Association between WHO First-Step Analgesic Use and Risk of Breast Cancer in Women of Working Age. Pharmaceuticals.

[59] Jaiswal V., Lee H.-J. (2023). Pharmacological Activities of Mogrol: Potential Phytochemical against Different Diseases. Life.

[60] Dang T.K., Hong S.-M., Dao V.T., Nguyen D.T., Nguyen K.V., Nguyen H.T., Ullah S., Tran H.T., Kim S.Y. (2023). Neuroprotective effects of total alkaloids fraction of Huperzia serrata on scopolamine-induced neurodegenerative animals. Phytother. Res..

[61] Bhat B.A., Almilaibary A., Mir R.A., Aljarallah B.M., Mir W.R., Ahmad F., Mir M.A. (2022). Natural Therapeutics in Aid of Treating Alzheimer’s Disease: A Green Gateway Toward Ending Quest for Treating Neurological Disorders. Front. Neurosci..

[62] Zhou S., Huang G. (2022). The biological activities of butyrylcholinesterase inhibitors. Biomed. Pharmacother..

[63] Dodson M., Shakya A., Anandhan A., Chen J., Garcia J.G., Zhang D.D. (2022). NRF2 and diabetes: The good, the bad, and the complex. Diabetes.

[64] Xia Y., Zhai X., Qiu Y., Lu X., Jiao Y. (2022). The Nrf2 in Obesity: A Friend or Foe?. Antioxidants.

[65] Wu S., Lu H., Bai Y. (2019). Nrf2 in cancers: A double-edged sword. Cancer Med..

[66] De Plano L.M., Calabrese G., Rizzo M.G., Oddo S., Caccamo A. (2023). The Role of the Transcription Factor Nrf2 in Alzheimer’s Disease: Therapeutic Opportunities. Biomolecules.

[67] Chen M.-m., Meng L.-h. (2022). The double faced role of xanthine oxidoreductase in cancer. Acta Pharmacol. Sin..

[68] Klisic A., Kocic G., Kavaric N., Jovanovic M., Stanisic V., Ninic A. (2020). Body mass index is independently associated with xanthine oxidase activity in overweight/obese population. Eat. Weight. Disord.-Stud. Anorex. Bulim. Obes..

[69] Prabhu S., Burrage E., Luthman J., Oudomvilay C., Luna N., Childers R., Hanshew A., Englund P., Tierno V., Hoover J. (2023). Xanthine oxidase mediates stress-induced Alzheimer’s Disease progression in triple transgenic mice. Physiology.

[70] Hasan M., Fariha K.A., Barman Z., Mou A.D., Miah R., Habib A., Tuba H.R., Ali N. (2022). Assessment of the relationship between serum xanthine oxidase levels and type 2 diabetes: A cross-sectional study. Sci. Rep..

[71] Huang J.-Q., Zhou J.-C., Wu Y.-Y., Ren F.-Z., Lei X.G. (2018). Role of glutathione peroxidase 1 in glucose and lipid metabolism-related diseases. Free Radic. Biol. Med..

[72] Shin E.-J., Chung Y.H., Sharma N., Nguyen B.T., Lee S.H., Kang S.W., Nah S.-Y., Wie M.B., Nabeshima T., Jeong J.H. (2020). Glutathione Peroxidase-1 Knockout Facilitates Memory Impairment Induced by β-Amyloid (1–42) in Mice via Inhibition of PKC βII-Mediated ERK Signaling; Application with Glutathione Peroxidase-1 Gene-Encoded Adenovirus Vector. Neurochem. Res..

[73] Kim Y.S., Gupta Vallur P., Phaëton R., Mythreye K., Hempel N. (2017). Insights into the Dichotomous Regulation of SOD2 in Cancer. Antioxidants.

[74] Massaad C.A., Washington T.M., Pautler R.G., Klann E. (2009). Overexpression of SOD-2 reduces hippocampal superoxide and prevents memory deficits in a mouse model of Alzheimer’s disease. Proc. Natl. Acad. Sci. USA.

